# ‘Like holding the axe on who should live or not’: adolescents’ and adults’ perceptions of valuing children’s health states using a standardised valuation protocol for the EQ-5D-Y-3L

**DOI:** 10.1007/s11136-022-03107-0

**Published:** 2022-02-24

**Authors:** Mimmi Åström, Helen Conte, Jenny Berg, Kristina Burström

**Affiliations:** 1 Health Outcomes and Economic Evaluation Research Group, Department of Learning, Informatics, Management and Ethics, Stockholm Centre for Healthcare Ethics, Karolinska Institutet, Tomtebodavägen 18 A, 171 77 Stockholm, Sweden; 2 Equity and Health Policy Research Group, Department of Global Public Health, Karolinska Institutet, Stockholm, Sweden; 3 Centre for Health Economics, Informatics and Health Services Research, Stockholm Health Care Services, Region Stockholm, Stockholm, Sweden; 4 Department of Neurobiology, Care Sciences and Society, Section of Nursing, Karolinska Institutet, Stockholm, Sweden

**Keywords:** Adolescents, Discrete choice experiment, EQ-5D-Y-3L, General population, Qualitative interviews, Time trade-off

## Abstract

**Purpose:**

There is an increasing interest for using qualitative methods to investigate peoples’ cognitive process when asked to value health states. A standardised valuation protocol for the EQ-5D-Y-3L instrument was recently developed. Little is known regarding how people think, reason, and feel when asked to value health states for children. The aim was to explore how adolescents and adults perceive the task of valuing children’s health states using the standardised valuation protocol.

**Methods:**

This was a qualitative study where adults (*n* = 10) and adolescents (*n* = 10) from the general population participated in individual video-interviews. Initially, participants reported their own health with the EQ-5D-3L instrument. Then they were asked to complete several valuations tasks for a 10-year-old child according to the standardised valuation protocol, followed by a semi-structured interview with open-ended questions to further explore participants’ perceptions. A qualitative content analysis was performed.

**Results:**

The two main categories that emerged from the data were ‘Thoughts and feelings when valuing children’s health states’ and ‘Strategies when valuing children’s health states’. Participants expressed feeling doubt, awfulness and being reluctant to trade-off life years, and questioned who has the right to value health states for children. Experience and point of view were strategies participants used to complete the valuation tasks.

**Conclusion:**

The findings from the present study can contribute to the understanding and interpretation of quantitative results where the standardised valuation protocol has been used to derive values for the EQ-5D-Y-3L. Furthermore, results of the study support the feasibility of including adolescents in valuation studies.

**Supplementary Information:**

The online version contains supplementary material available at 10.1007/s11136-022-03107-0.

## Introduction

There are several instruments for measuring health-related quality of life (HRQoL) among children and adolescents [1]. One of these instruments is the EQ-5D-Y-3L, a generic instrument developed to measure self-reported health among children from the age of eight [2, 3]. The first part of the instrument, the descriptive system, covers five dimensions of health (mobility; looking after myself; doing usual activities; having pain or discomfort; feeling worried, sad or unhappy) with three severity levels. By combining the dimensions and the severity levels, 243 unique health states can be formed. For example, health state 33,333 represents ‘a lot of problems’ in all dimensions. The second part is a visual analogue scale (EQ VAS), where the respondent rates his/her overall health between 0 (worst imaginable health) and 100 (best imaginable health) [2].

HRQoL instruments can be used to combine length of life and HRQoL into quality-adjusted life years (QALYs). Each of the health states is converted into a single value, using valuation methods such as the time trade-off (TTO), the standard gamble (SG) and the visual analogue scale (VAS) [4]. Eliciting values from children themselves or from a proxy have yielded different results, where the magnitude depends on for example valuation method and the health states valued [5]. To guide the elicitation of values for the EQ-5D-Y-3L instrument, a standardised protocol valuation protocol has been published, which includes the valuation methods TTO and discrete choice experiment (DCE) [6]. In the TTO method, respondents are asked to choose, for a 10-year-old child, between being in a specified health state for X number of years and a shorter time in full health. Using DCE, respondents are asked to choose between two health states which they prefer for a 10-year-old child. Two value sets have been published using this standardised valuation protocol for the EQ-5D-Y-3L, from Slovenia [7] and Japan [8]. In both value sets, adults valued pain or discomfort as the most important health dimension for a child.

There are several methodological and practical challenges concerning valuation of health states for adults, and these are even more pronounced when it comes to children’s health states. Central considerations are whether to derive health state values by asking respondents to value their own health state (experience-based) or to value described health states (hypothetical) and choosing which valuation methods to use [9, 10]. Previous methodological studies have shown that using the TTO and DCE methods have resulted in higher mean values, i.e., same health states being less severe, for children compared to adults [11]. Lipman et al. [12] point on a methodological issue also when valuing hypothetical health states: the individual perspective, as in the protocol for the adult versions of the EQ-5D, where respondents should think of ‘someone like you’ being in the hypothetical health states, and the child perspective, as in the protocol for the EQ-5D-Y-3L, i.e., to value health for ‘someone else’, a 10-year-old [12].

The need to investigate the cognitive process people go through when valuing health states was recognised in the first valuation study of the adult version EQ-5D-3L [13], and the burden on respondents and the complexity of the tasks have been emphasised [14, 15]. Considering alternative approaches, and hence future updates of the standardised valuation protocol might be of importance, e.g., regarding the relevance of using values from adults or younger age groups for the valuation of children’s health states [8]. Using qualitative methods when planning and organizing valuation exercises is a key element [16]. Results from qualitative studies might therefore contribute with insights that could support further development of the standardised protocol. There are few qualitative studies investigating peoples’ thoughts and feelings when valuing health states for children, especially in relation to the standardised valuation protocol [17] and few studies addressing the difference between child and adult perspective [12]. Hence, the aim of the present study was to explore how adolescents and adults perceive the task of valuing children’s health states using the standardised valuation protocol for the EQ-5D-Y-3L.

## Materials and methods

### Study design

A qualitative study was carried out where adults and adolescents from the Swedish general population participated in individual interviews to explore how they perceived the task of valuing health states according to the standard valuation protocol for the EQ-5D-Y-3L instrument. A qualitative content analysis was performed [18]. The Consolidated criteria for Reporting Qualitative research (COREQ) checklist was applied [19] (Online Resource Appendix 1).

### Setting and study participants

Recruitment of participants was based on convenience sampling [20] and participants were enrolled on a first come, first served basis. Adults were recruited by advertising the study on Karolinska Institutet’s webpage, and adolescents by sending an e-mail to all students aged 15 years or older in a school in Stockholm. Adults and adolescents who replied received a letter of information and a form for informed consent. Participants had to speak and understand Swedish, which was assessed by the interviewer in the beginning of each interview.

For the main data collection, 20 individual interviews were conducted: ten adults (90% females; median age 39.5; median EQ VAS score 82.5) and ten adolescents (70% females; median age 17; median EQ VAS score 77.5). Background characteristics of study participants, parental status, participants’ experience with children, and how they reported their own health with the EQ-5D-3L and EQ VAS are shown in Table 1.

**Table 1:** Background characteristics study participants

Participant	Sex	Age	EQ-5D-3L health state	EQ VAS score	Experience of children*	Being a parent
P1	Female	17	11222	60	Yes	
P2	Female	17	11111	85	Yes	
P3	Male	17	11111	85	Yes	
P4	Female	16	11111	70	Yes	
P5	Female	17	11112	95	Yes	
P6	Male	18	11111	80	Yes	
P7	Male	17	11112	75	Yes	
P8	Female	16	11122	75	Yes	
P9	Female	16	11112	80	Yes	
P10	Female	16	11112	75	Yes	
P11	Female	42	11111	90		No
P12	Female	43	11111	80		Yes
P13	Female	24	11111	90		No
P14	Female	41	11111	70		Yes
P15	Male	47	11111	90		Yes
P16	Female	22	11222	60		No
P17	Female	33	11112	50		Yes
P18	Female	26	11122	85		No
P19	Female	40	11111	90		Yes
P20	Female	39	11111	80		Yes

*For example baby sitting or organising activities for children

### Data collection

Pilot interviews were conducted (Online Resource Appendix 2). The main data collection took place between February and April 2021. The first author, who had previous experience with interviewing children [21] and adults, conducted the interviews which lasted between 44 and 79 min.

The interview began with the participant reporting their own health with the EQ-5D-3L. This was followed by the completion of the valuation tasks according to the standardised valuation protocol [6]. The interviewer and the participants had their video cameras on during the interview through Zoom to facilitate communication and the interviewer’s screen was shared to visualise the tasks. Participants were asked to confirm all choices made in the valuation tasks. The latter part of the interview was semi-structured, with identical open-ended questions to both groups (Online Resource Appendix 3). Ethical approval was granted by the Swedish Ethical Review Authority (Dnr: 2020-03753, 2020-05390).

### Data analysis

**Fig. 1:**
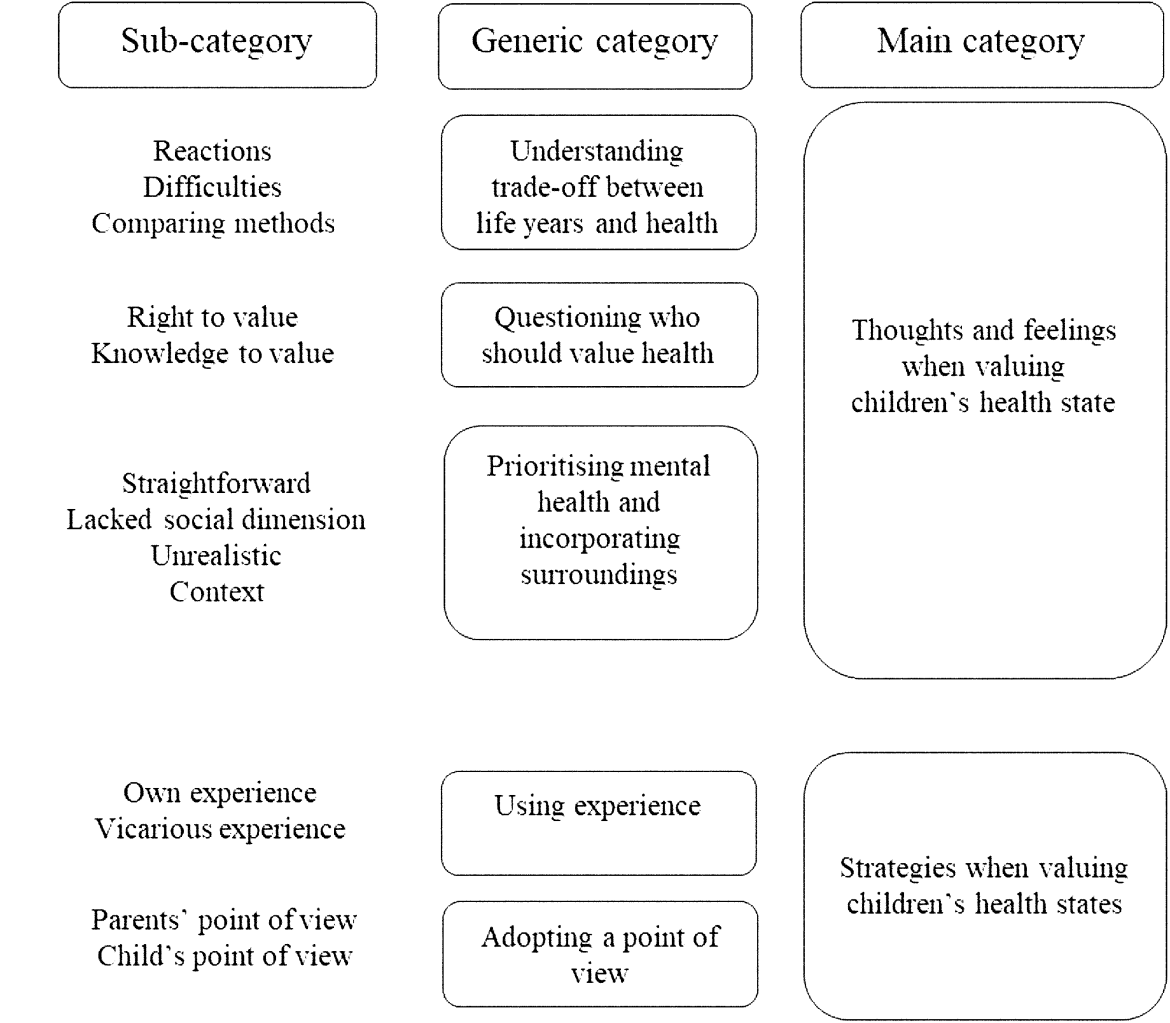
Illustration of the process of moving from sub-categories to generic and main categories

All interviews were recorded and transcribed verbatim. An inductive qualitative content analysis in NVivo 1.4.1 was performed, where categories were derived from the raw data (from codes to sub-, generic- and main categories), guided by three phases: preparation, organising and reporting [18]. The process of moving from sub-categories to generic categories and finally to main categories is illustrated in Fig. 1 (details in Online Resource Appendix 4).

## Results

Two main categories emerged from the data: ‘Thoughts and feelings when valuing children’s health states’ and ‘Strategies when valuing children’s health states’ (Fig. 1). These categories were intertwined, as how adolescents and adults thought and felt influenced what strategies they used and the other way around. See Online Resource Appendix 5 for quotes in addition to those below.

### Main category 1: thoughts and feelings when valuing children’s health states

This main category contained three generic categories. The level of emotional involvement varied and a variety of thoughts and feeling arose among the participants depending on the severity of the health state valued and the valuation method.

#### Understanding the trade-off between life years and health

In general, participants understood quickly that their task was to trade between quantity and quality of life in the TTO task.

‘Yes, it becomes more black and white. Compared to when you thought for yourself, then you reasoned… you avoid having this pain… But here, it just becomes these years we take away from the child’s life’ R12, adult

Many of the participants sought the interviewer’s confirmation regarding the fact that both Life A and Life B are followed by death in this task, and it was clear that most participants thought this was offensive. Valuing health for a 10-year-old child was brought up by most participants as something that made the tasks difficult.

‘Except from feeling a bit horrible at times. It felt like, God, what a horrible thought, but if I have to choose, I choose like this. So, I think it was… it was interesting. It was fun to… get an eye-opener’

R13, adult

‘Oh. You feel so grotesque if you think about [shortening] a 10-year-old’s life’ R10, adolescent

Although, most of the participants expressed these feelings, a majority traded life years for the more severe health states. A few participants argued that avoiding suffering for the child was most important, no matter how short the life for that child became. It was also clear that the initial reaction, i.e., unwillingness to trade-off life years, did not necessarily result in non-trader behaviour.

‘…when it concerns a child, it is almost like you get a bad conscience to cast away life, and this might not have been the case if it had been an adult. So, for example, if it had been for your partner it might also have been easier compared to a child, actually’ R20, adult

One specific trigger observed among most of the participants was the confirmation box in the TTO task. When they reached the point of indifference between Life A and Life B, the confirmation box appears with the following text ‘Your response suggests that to avoid a 10-year-old child being in this health state for 10 years you are willing to give up X year(s)’.

After understanding the idea behind the tasks, many reacted to the valuation tasks and especially the TTO task. Most participants expressed being uncomfortable when making the decision, especially shortening the life for the child. It was also clear that participants felt a responsibility when completing the tasks. Participants also expressed feeling horrible and grotesque when completing the TTO task. One participant reacted to the tasks by being worried over how the valuations were going to be used in practice.

‘No, but I thought like this, if you are going to use this for something, physicians or others who will receive this answer and then just: “yes but when to end someone’s life”, but I thought if that will be based on this, because I hope not’ R14, adult

Most of the participants thought that the DCE task was easier to complete compared to the TTO task, foremost because they did not have to shorten the life for a child in the DCE task. Many also recognised the DCE to be more theoretical and more straightforward.

#### Questioning who should value health

Many of the participants reflected over who has the right to value health for a 10-year-old child, and most found it hard and expressed doubt. Even though participants expressed these feelings and thoughts, all managed to complete the tasks.

‘Why should I… decide which of these… what right do I have to decide, which of these are better or worse, that is what is happening in my brain. Hmm, why should anyone except that child or that parent have the right to say what is better for them?’ R1, adolescent

Not having experience of the health state to be valued, implying not being the right person to value it, was expressed by many. Participants brought up that it is impossible to imagine how a health state is for another person and therefore hesitated to assign a value. Many expressed concerns about completing the valuation in an accurate way. A common reflection was that they would have preferred to value their own health state over valuing health states for someone else.

‘…I cannot identify with these health conditions, I do know people who have had these conditions, but I myself cannot imagine how that would be. So, it is really difficult for me to value these things when I have not experienced it myself’ R1, adolescent

#### Prioritising mental health and incorporating surroundings

To value health states based on the dimensions and the severity levels in the EQ-5D-Y-3L instrument was in general accepted by the participants.

‘I thought it was good. At least I got a picture of what I thought it [the health state] would sort of imply’ R17, adult

Many reflected over the division of health in physical and mental health. Most participants described mental health aspects and, hence, the dimension feeling worried, sad, or unhappy as most important. Some participants even thought that mental health outweighs all physical health problems.

‘I consider the psychological aspect more, because I feel that psychological problems are much heavier, as that is the root of life’ R4, adolescent

In contrast, one participant reflected over the importance for the individual not to lose their integrity, and therefore thought that the dimension looking after myself was very important. A variation in awareness of one’s views among the participants was observed: some knew that they thought mental aspects were most important; others were surprised about their prioritisation.

Pain was also pointed out as an important dimension. Many expressed that they would not like to see anyone having pain, and some stressed that even more strongly when valuing health for a 10-year-old child. Being unhappy in combination with pain was considered as the worst by several.

‘Yes, basically I remove the first three [dimensions]. The relationship pain and psychological pain, the rest I find is not so important in this context’ R14, adult

Some recognised some health states as implausible. Furthermore, many of the participants struggled and found it unrealistic that the health states were constant in the TTO task as there was no chance for the child to improve. Many participants mentioned social aspects as an important part of life and that they would have liked such a dimension in the EQ-5D-Y-3L.

‘It [a social dimension] would have been something one would want to include, as the social part is quite important’ R20, adult

Participants reflected over the context the imagined 10-year-old child lived in and how that affected the relative importance of health dimensions. The child would have different challenges when coping with different health states depending on the environment.

Participants thought it to be important whether the child had social support, family, and a good situation in school. Some argued that some of the health states would be even worse for adults compared to a child, as parents/guardians might make it possible to cope with some of the health states if they occur when you are 10-year-old.

‘I think it is more difficult for adults to need this support than for children, as children often have their parents present and hmm, and I think it is more difficult for adults to get that kind of help and support’ R1, adolescent

### Main category 2: strategies when valuing children’s health states

This main category covered two generic categories. The strategies used by the participants to complete the valuation tasks varied between and within participants. Depending on the health state to be valued or the valuation task, TTO or DCE, some participants employed different strategies while others stuck to the same strategy.

#### Using experience

Participants used own experience and vicarious experience as strategies to seek understanding and knowledge about the health state. They reflected over their own current health, for example if they had a disease with similar features.

‘Since I myself have been suffering from mental illness during a long period, I would have preferred this short [option]. I would have preferred a shorter life and avoiding… Because even so, you do not want to do anything if you feel so bad. Then I prefer to remove that and to have a better shorter time instead of a longer period that is just hard’

R10, adolescent

A variation was observed among participants: some reflected on their own time as a 10-year-old child; others reflected more generally on different health states they had experienced regardless of age. Participants were also seeking understanding by reflecting on health states people in their surroundings experienced, referred to as vicarious experience. The person they referred to was for example a family member, a relative, someone they had heard of, or even a movie star.

‘Because I saw that movie about Stephen Hawking on Netflix, and it is beautiful, and he had many physical problems, but he did have a really good life’ R14, adult

#### Adopting a point of view

Participants reflected on whose point of view to adopt when completing the valuation tasks, and how adopting different point of views might yield different values. There was no clear pattern regarding how the actual valuation of the health state was affected by which point of view was adopted, as it was not clear which point of view had been taken for valuing given health states.

The parents’ point of view was mentioned by most of the participants: some argued that from an egoistic point of view, as a parent (regardless of being a parent or not), you would like your child to live as many years as possible; others emphasized the importance of avoiding suffering for your child. Participants reflected both on how the health state for the child would affect the parent and on how a parent would have valued the health state for their own child. Different points of view were used as strategies when valuing. Awareness of how the participants’ decisions were affected by emotional ties was shown by some, especially when imagining their own child.

‘…if it was my own children, then I would like them to live, to maximise time, one is selfish with it. But if you think from the person’s own perspective, then I would say no – why should you live and just suffer?’ R17, adult

‘I tried to think about how I would do if I thought logically. But if it was my own child, logic alone would not matter’ R2, adolescent

Many participants reflected over the different viewpoints that could be adopted but concluded that they could only use their own viewpoint in completing the valuation tasks. Some also recognised that they would have preferred to ask the child him- or herself about their preferences.

## Discussion

This interview study provides insights into how adults and adolescents from a general population perceived the task of valuing health states for a 10-year-old child according to the standardised valuation protocol for the EQ-5D-Y-3L instrument [6]. Participant’s thoughts and feelings influenced the strategies they used, and vice versa. The level of emotional investment was continually changing among some participants depending on the severity of the health state to be valued or which valuation method being used, while others remained in the same emotional state throughout the valuation tasks.

It was clear that participants found the TTO task most offensive and were uncomfortable with completing it, as it incorporates a trade-off between life years and health for a child. Participants would have preferred to value health for themselves or another adult instead. The importance of avoiding suffering for a child was one of the motivations raised by several participants on why they nevertheless ended up trading-off life years.

Participants’ ability to quickly grasp the purpose of trading-off between life years and health is in line with a previous study [22]; in our study, no difference between adults and adolescents was observed regarding understanding the tasks. Hence, our results support the engagement of adolescents from 16 years in completing TTO tasks. Previous studies have shown that adults’ and adolescents’ preferences differ [23, 24] and, therefore, our results are important as they demonstrate the feasibility of capturing adolescents’ values, even though we did not investigate possible differences in values for the health states in our study. When asked to share their thoughts and feelings regarding the valuation tasks, many adolescents and adults expressed feelings of reluctance and doubt when completing the TTO tasks, as reported earlier [15]. Future studies could address potential differences between males and females which was not possible with our convenience sampling.

Valuing health states by the EQ-5D-Y-3L instrument was generally accepted by participants. However, many participants expressed that the EQ-5D-Y-3L did not cover all dimensions important to health, which has been recognised earlier [25].

In the present study, it was clear that mental health was the most important dimension when valuing health. This is in line with previous general population studies where adults and adolescents valued their own health [26–28]. Mental and cognitive health were also more important than physical health in a recent study using the TTO [21]. In our study, some participants even pointed out the mental health aspects as paramount, hence, focused only on this dimension. Exclusively making the valuation decision on solely one health dimension has been observed earlier [29]. Participants identifying implausible health states, as in our study, was also found in the study by Karimi et al. [15]. The context the child was growing up in and the social support system, for example support by parents or guardians, were central in our study and has previously been identified as important [15]. Participants reflected over that some health states might even be easier for children than adults to cope with, as children commonly have support by an adult for example with problems in the dimension looking after myself. Social aspects are incorporated in the dimension doing usual activities, but participants wanted social aspects to be a specific dimension.

The question regarding who should value health states is a burning topic, discussed in the literature and recognised as a normative issue [1, 30, 31]. In the standardised valuation protocol [6], the choice of having an adult general population valuing described (hypothetical) health states was mainly motivated by the ‘taxpayer’ argument. The main difference between how values for hypothetical health states are derived for the adult version of the EQ-5D-5L [32] and the EQ-5D-Y-3L [6] is the shift of perspective, from individual to child perspective, as pointed out by Lipman et al. [12]. This change of perspective might, in itself, lead to differences in derived values.

The strategy of using experience to concretise the health state to be valued is recognised from previous studies [15, 22] as is focusing on the future when using the TTO [33, 34]. In the present analysis, the categorization of different types of experience into own and vicarious experience was influenced by Cubi-Molla et al. [35]. Even though it was clearly stated in our study that they were asked to value health states for a 10-year-old child, adopting different point of views was a strategy many participants used.

The location for the interview might affect data, and it is important that the participants feel comfortable [36]. That many participated from home could potentially have enabled participants to express themselves more. Advantages, in terms of increased participation and flexibility, and disadvantages, in terms of loss of visual cues and difficulty in building a positive atmosphere, have been recognised using digital aids such as Zoom for interviewing [37, 38]. Weak internet connection was sometimes a challenge in our study. To validate data in the present study the interviewer asked for confirmation that she had understood the participants in a correct manner [38]. A limitation of our study was using the strategy of first come first served when enrolling participants, this could possibly have influenced the results as it resulted in the inclusion of few (*n* = 4) males. However, to the best of our knowledge, there are no previous literature showing differences between males and females when it comes to how people perceive valuing health states for children. Future research investigating differences between males and females are warranted. In our study, all adolescents had experience with children and a majority of adults were parents, this might have influenced how the perceived the task of valuing health states for a child.

It is important to reflect on how the results from our study can be transferred to and valid for other settings and populations [36, 39, 40]. In our study, the context, sample strategies, sample size, participants, and procedures are described thoroughly to enable the reader to form an opinion regarding transferability [41]. However, as the EQ-5D-Y-3L is an international instrument, the questions asked in this study are most likely relevant for other contexts where the instrument is being used. To enhance credibility, a sample of the transcribed material was coded individually by first and the second author, discussed and compared. Quotes from participants increase confirmability. To promote dependability, activities and decisions were documented. Some qualitative studies have previously examined psychometric properties of the EQ-5D [42–44], but this is, to the best of our knowledge, the first study investigating what lies behind the numbers when valuing health states according to the standardised valuation protocol for the EQ-5D-Y-3L. Results from the present study can increase understanding of how adolescents and adults perceive completing such valuations and encourage similar studies in the protocol development and when planning full-scale valuation studies. Furthermore, this study provides important knowledge regarding how emotional strenuous these valuation tasks were for the respondent and raises the question if this is how we want people to react when valuing health state for children. Differences between the child perspective and own perspective need to be thought of and addressed when applying similar approaches in the standardised valuation protocol for EQ-5D-Y-3L as for the valuation protocol for the adult version EQ-5D-5L.

## Conclusion

Thoughts and feelings were explored, and strategies revealed. Although adolescents and adults managed to complete the valuation tasks to derive values for the EQ-5D-Y-3L, doubt, awfulness and being reluctant to trade-off life years were commonly expressed feelings. Participants questioned who has the right to value health states for someone else and recognised their own limitations in judging health states unfamiliar to them. Experience and point of view were used as strategies to complete the valuation tasks. These findings can contribute to the need of further developing the standardised valuation protocol for the EQ-5D-Y-3L as well as understanding and interpretating the quantitative results.

## Supplementary Information

Below is the link to the electronic supplementary material.

Supplementary file1 (DOCX 35 kb)

## Data Availability

Data sharing is not possible according to Swedish law.
