# Supplementary material for: ‘Like holding the axe on who should live or not’: adolescents’ and adults’ perceptions of valuing children’s health states using a standardised valuation protocol for the EQ-5D-Y-3L
Source: Qual Life Res. 2022 Feb 24;31(7):2133–42. doi: 10.1007/s11136-022-03107-0 (PMC9188517; doi:10.1007/s11136-022-03107-0)
Supplement: Supplementary file 1 — Supplementary file1 (DOCX 35 kb) [file 11136_2022_3107_MOESM1_ESM.docx]

**Electronic Supplementary Material (ESM)**

**Title: ‘Like holding the axe on who should live or not’
- Adolescents’ and adults’ perceptions of valuing children’s health states using a standardised valuation protocol for the EQ-5D-Y-3L**

**Journal: Quality of Life Research, 2021**

CONTENTS

[Appendix 1 – COREQ Checklist 2](#_Toc81742786)

[Appendix 2 – Pilot interviews, preparation of material 4](#_Toc81742787)

[Appendix 3 – Interview guide 5](#_Toc81742788)

[Appendix 4 – Data analysis process 6](#_Toc81742789)

[Appendix 5 – Additional quotes from participants 7](#_Toc81742790)

# Appendix 1 – COREQ Checklist

**COREQ (COnsolidated criteria for REporting Qualitative research) Checklist**

A checklist of items that should be included in reports of qualitative research. You must report the page number in your manuscript where you consider each of the items listed in this checklist. If you have not included this information, either revise your manuscript accordingly before submitting or note N/A.

| **Topic** | **Item No.** | **Guide Questions/Description** | **Reported on Page No.** |
| --- | --- | --- | --- |
| **Domain 1: Research team and reflexivity** |  |  |  |
| *Personal characteristics* |  |  |  |
| Interviewer/facilitator | 1 | Which author/s conducted the interview or focus group? | 6 |
| Credentials | 2 | What were the researcher’s credentials? E.g. PhD, MD | 6 |
| Occupation | 3 | What was their occupation at the time of the study? | 6 |
| Gender | 4 | Was the researcher male or female? | 14 |
| Experience and training | 5 | What experience or training did the researcher have? | 6 |
| *Relationship with participants* |  |  |  |
| Relationship established | 6 | Was a relationship established prior to study commencement? | 6 |
| Participant knowledge of the interviewer | 7 | What did the participants know about the researcher? e.g. personal goals, reasons for doing the research | N/A |
| Interviewer characteristics | 8 | What characteristics were reported about the inter viewer/facilitator? e.g. Bias, assumptions, reasons and interests in the research topic | 6 |
| **Domain 2: Study design** |  |  |  |
| *Theoretical framework* |  |  |  |
| Methodological orientation and Theory | 9 | What methodological orientation was stated to underpin the study? e.g.  grounded theory, discourse analysis, ethnography, phenomenology, content analysis | 6 |
| *Participant selection* |  |  |  |
| Sampling | 10 | How were participants selected? e.g. purposive, convenience, consecutive, snowball | 6 |
| Method of approach | 11 | How were participants approached? e.g. face-to-face, telephone, mail, email | 6 |
| Sample size | 12 | How many participants were in the study? | 6 |
| Non-participation | 13 | How many people refused to participate or dropped out? Reasons? | 6 |
| *Setting* |  |  |  |
| Setting of data collection | 14 | Where was the data collected? e.g. home, clinic, workplace | 6 |
| Presence of nonparticipants | 15 | Was anyone else present besides the participants and researchers? | 6 |
| Description of sample | 16 | What are the important characteristics of the sample? e.g. demographic data, date | 6 |
| *Data collection* |  |  |  |
| Interview guide | 17 | Were questions, prompts, guides provided by the authors? Was it pilot tested? | Appendix 3 |
| Repeat interviews | 18 | Were repeat inter views carried out? If yes, how many? | N/A |
| Audio/visual recording | 19 | Did the research use audio or visual recording to collect the data? | 6 |
| Field notes | 20 | Were field notes made during and/or after the interview or focus group? | Appendix 4 |
| Duration | 21 | What was the duration of the inter views or focus group? | 6 |
| Data saturation | 22 | Was data saturation discussed? | N/A |
| Transcripts returned | 23 | Were transcripts returned to participants for comment and/or  correction? | N/A |
| **Topic** | **Item No.** | **Guide Questions/Description** | **Reported on Page No.** |
| **Domain 3: analysis and findings** |  |  |  |
| *Data analysis* |  |  |  |
| Number of data coders | 24 | How many data coders coded the data? | Appendix 4 |
| Description of the coding tree | 25 | Did authors provide a description of the coding tree? | Figure 1 |
| Derivation of themes | 26 | Were themes identified in advance or derived from the data? | 6 |
| Software | 27 | What software, if applicable, was used to manage the data? | 6 |
| Participant checking | 28 | Did participants provide feedback on the findings? | N/A |
| *Reporting* |  |  |  |
| Quotations presented | 29 | Were participant quotations presented to illustrate the themes/findings?  Was each quotation identified? e.g. participant number | 8-11 |
| Data and findings consistent | 30 | Was there consistency between the data presented and the findings? | 8-11 |
| Clarity of major themes | 31 | Were major themes clearly presented in the findings? | 8-11 |
| Clarity of minor themes | 32 | Is there a description of diverse cases or discussion of minor themes? | 8-11 |

Developed from: Tong A, Sainsbury P, Craig J. Consolidated criteria for reporting qualitative research (COREQ): a 32-item checklist for interviews and focus groups. *International Journal for Quality in Health Care*. 2007. Volume 19, Number 6: pp. 349 – 357

**Once you have completed this checklist, please save a copy and upload it as part of your submission. DO NOT** **include this checklist as part of the main manuscript document. It must be uploaded as a separate file.**

# Appendix 2 – Pilot interviews, preparation of material

The standardised valuation protocol was translated into Swedish by the authors (MÅ, KB, JB). Initially, the first (MÅ) and the last (KB) authors constructed an interview guide (Online Resource Appendix 3) that was tested during three pilot interviews with adults and two pilot interviews with adolescents. MÅ had previous experience of conducting interviews with both children and adolescents as well as adults. All interviews were recorded to enable the interviewer to focus on the interaction during the conversation (Kvale & Brinkmann, 2015). MÅ performed the interviews and the other authors listened to recordings, reviewed the interview technique, understandability, and construction of the guide. Discussions amongst the authors led to some changes in the setup of the interviews, for example, that key words in the interview guide needed to be even more emphasized and the anticipated break needed to be flexible. The pilot interviews also provided practical insights, for example how to conduct valuation tasks through Zoom and how to get the best audio quality.

In the preparation phase sample size was discussed and information power was considered to guide this decision. It was difficult to anticipate how much information the participants could provide on this topic as this was their first experience with valuing health states for children using these methods. The aim of the study can be considered quite narrow which imply the need of less participants. The number of interviews was continuously considered and discussed in terms of saturation among the co-authors during the process of the study.

Kvale S, Brinkmann S. Interviews. Learning the Craft of Qualitative Research Interviewing. 2015. Thousand, Oaks, CAL: Sage

# Appendix 3 – Interview guide

1. How did you think about this?
2. Can you tell me more about how you perceived this valuation task (show a picture of the TTO task to help the interviewee to remember)

- Thought, reasoned, felt

1. Can you tell me more about how you perceived this valuation task (show a picture of the DCE task to help the interviewee to remember)

- Thought, reasoned, felt

1. Can you tell me how you perceived the different tasks?

- Thought, reasoned, felt

1. Can you describe how it was to perform these valuation tasks for a 10-year-old child?

- Thought, reasoned, felt
- An adult instead of a 10-year-old child

1. Can you tell me how you perceived the description of the health states that were used in the valuation tasks? (show a picture of a health state)

- Focused on something specific

1. What would you like me to take back to those who design these standardised protocols for valuation tasks?

- Advice, potential changes

**Probing questions to use during the interview**

- Can you tell me more?
- How do you mean?
- Could you give an example of what you mean?
- Why?
- How did you feel then?

**Questions before finalising the interview**

- What did you think about this interview?
- Is there something you would like to add?
- Thank you for taking your time to talk to me!

# Appendix 4 – Data analysis process

**The following details can be read to increase the understanding of the data analysis process.**

Alongside the valuation tasks, answers given by participants and observations made by the interviewer were documented by hand and later transferred to Excel.

In the preparation phase, the authors strive at making sense of the data as a whole and this was done by repeatedly reading and listening to the material. This was continuously done while the data collection was ongoing. The process of coding data started after all interviews were conducted. The first author transcribed a sample of the recorded interviews, and all transcribed material was read through repeatedly. This was an opportunity to form a first opinion of the material and start the process of analysing the data. A sample of the audio files and the transcribed material was shared and discussed among all authors. In the organising phase, the first step was to write down headings and notes in the margin of the transcribed material. This was an ongoing process where new headings and notes occurred or were removed as the work continued. A draft of this open coding was shared and discussed with the second author. The last step in the organising phase is the abstraction process, which means formulating a description of the topic by producing categories. The specific codes were organized into broader sub-categories, these sub-categories incorporated both similar and dissimilar content. The sub-categories where thereafter grouped into generic categories and further grouped into main categories. The categorization was carried out based on the first author’s interpretation regarding what belonged to the same category, but each step was discussed among all authors. In the reporting phase, the focus was on the process of reporting how the data was analysed, and the description of the results.

# Appendix 5 – Additional quotes from participants

**Additional quotes from participants belonging to the following generic categories.**

Understanding the trade-off between life years and health

*’I think that, no, then I think, why not, so, it is not bad to have a short life, no, then I rather chose five years compared to this suffering that is ahead’ R17, adult*

*‘To give up, it sounds so bloody brutal. It sounds like… one should take someone’s life. I don’t know, it is a strange phrasing.’ R6, adolescent*

*‘In the second task [DCE] you received more information, and both children had some kind of health condition, and I thought that was much easier to compare […] It contained more details regarding both children. Which I thought made it much easier to choose’ R13, adult*

Prioritising mental health and incorporating surroundings

*‘Most often it is a lot about mental aspects in how you value a life, I find.’ R14, adult*

*‘I think more that integrity is very important. And when you lose that, your integrity, it feels undignified for the person. That carries quite a lot of weight. That you should have the right to have your own bubble and your own sphere’ R6, adolescent*

*’Just that I prioritised mental health more than I thought I would. That was something that I reflected on’ R5, adolescent*

*‘It was interesting, because I think you realize very quickly what you value as more important. And I think I have a tendency to value the psychological part more […] and there I think I have a tendency to almost overvalue it, or at least, I hold it high as a priority’ R16, adult*

*’Yes, that is really difficult, as it depends on so many things, and if there is any opportunity of improvement. In real life, you never know what will happen’ R14, adult*

*‘But regarding better and worse, I think that depends a bit on how society supports you’ R4, adolescent*

Point of view

*‘If you are thinking that you have a child’s life in your hands, and you have to decide what is best for someone else. It was only possible to use your own values, what would I have chosen. Had it been a real child I would have asked: what do you want?, if that had been possible. So that was probably the difficult part, to imagine that I somehow should decide for someone else, especially when it’s a child.’ R10, adolescent*
